# Fuzzy Ontology-Based System for Driver Behavior Classification

**DOI:** 10.3390/s22207954

**Published:** 2022-10-19

**Authors:** Susel Fernandez, Takayuki Ito, Luis Cruz-Piris, Ivan Marsa-Maestre

**Affiliations:** 1 Universidad de Alcalá, Departamento de Automática, Escuela Politécnica Superior, Campus Universitario, Ctra. Madrid-Barcelona, Km. 33,600, 28805 Madrid, Spain; 2 Department of Social Informatics, Kyoto University, Kyoto-shi 606-8501, Japan

**Keywords:** driver behavior, fuzzy rule-based system, classification, knowledge, sensor networks

## Abstract

Intelligent transportation systems encompass a series of technologies and applications that exchange information to improve road traffic and avoid accidents. According to statistics, some studies argue that human mistakes cause most road accidents worldwide. For this reason, it is essential to model driver behavior to improve road safety. This paper presents a Fuzzy Rule-Based System for driver classification into different profiles considering their behavior. The system’s knowledge base includes an ontology and a set of driving rules. The ontology models the main entities related to driver behavior and their relationships with the traffic environment. The driving rules help the inference system to make decisions in different situations according to traffic regulations. The classification system has been integrated on an intelligent transportation architecture. Considering the user’s driving style, the driving assistance system sends them recommendations, such as adjusting speed or choosing alternative routes, allowing them to prevent or mitigate negative transportation events, such as road crashes or traffic jams. We carry out a set of experiments in order to test the expressiveness of the ontology along with the effectiveness of the overall classification system in different simulated traffic situations. The results of the experiments show that the ontology is expressive enough to model the knowledge of the proposed traffic scenarios, with an F1 score of 0.9. In addition, the system allows proper classification of the drivers’ behavior, with an F1 score of 0.84, outperforming Random Forest and Naive Bayes classifiers. In the simulation experiments, we observe that most of the drivers who are recommended an alternative route experience an average time gain of 66.4%, showing the utility of the proposal.

## 1. Introduction

Nowadays, information and communication technologies are being applied in an increasing variety of fields for the benefit of modern society, and one of these fields is road transportation [1]. Intelligent Transportation Systems (ITS) allow data exchange and synchronization among different systems, including infrastructure, vehicles, users, traffic management, and weather information systems.

One of the most relevant elements impacting transport safety is driver behavior. Several studies have concluded that more than 90% of traffic accidents are due to driver mistakes [2]. For that reason, the process of analyzing and modeling human driver behavior as realistically as possible is a very active research topic, with the goal of improving the safety in road transportation.

*Driving style* is a complex concept influenced by many factors that makes it difficult to define precisely. This has led to numerous terms, often lacking an agreed definition [3]. *Driving events* are generally understood as maneuvers that occur during the driving tasks, such as acceleration, deceleration, turning, and lane changes, which can be used to identify the driving style [4]. The *driving pattern* is closely related to road type, weather conditions, or driving conditions and driving style [5] but does not include that information specifically. In this work, *driver behavior* is defined as the set of intentional/unintentional characteristics and actions that a driver performs while driving a vehicle. Depending on a variety of elements such as driving experiences, emotions, and driving preferences, among others [6], drivers can exhibit different types of behavior. These can range from passive behavior, characterized by low speed and little risk, to aggressive behavior, characterized by riskier actions while driving. They may also have certain behaviors that indicate poor physical condition, such as fatigue or illness.

Along with the early detection of abnormal driver behavior, the classification of driver behavior into different profiles can be very useful for Intelligent Transportation Systems. Driving assistance systems can generate alerts when they detect abnormal behaviors and also give the drivers advice, considering their profile, preventing unwanted situations such as accidents and traffic jams.

A wide variety of input data can be used to perform the automatic classification of driver behavior into different profiles, taking into account the factors that influence driver behavior. This includes driver-related, vehicle-related, and environment-related factors.

The current state-of-the-art for driver behavior classification presents a number of limitations, mainly the lack of expressiveness and the difficulty of working with the uncertainties in the environment. We discuss these limitations in Section 2. To close this gap, we proposes a fuzzy system for driver classification into different profiles depending on their behavior. The main contributions and results of this work are as follows:

- Construction of a driver behavior model based on data from a realistic simulator and creation of an ontology for knowledge representation according to that model. This ontology has been integrated with a traffic ontology. The whole knowledge base allows us to model different general traffic scenarios, with driver-behavior-related knowledge.
- A fuzzy logic architecture based on the Mamdani approach that allows us to classify drivers into five profiles (*very passive*, *passive*, *normal*, *aggressive*, and *dangerous*), taking into account driver-related parameters such as age, gender, speed, and accelerator and brake usage. The proposed system has the following particularities regarding the knowledge base and the inference engine:
  1. The knowledge base is made up of the driver behavior ontology connected to a traffic ontology, a database from a driving simulator, and a rule base learned through a genetic algorithm.
  2. The proposed inference engine allows us to deal with the absence of data by substituting the membership degrees of the absent variables during the inference process, taking advantage of the properties of the conjunction operator.
- An experimental evaluation to validate the effectiveness of the classification system, the expressiveness of the ontology, and the utility of the classification system in an ITS scenario. To compare the results of the proposed system with other approaches, two alternative classification algorithms have been implemented: a Naive Bayes classifier and a Random Forest classifier. The fuzzy classifier clearly outperforms the other approaches, mainly due to its more appropriate handling of uncertainty. In addition, experiments show that the proposed knowledge model is expressive enough for the evaluated scenarios, achieving good results in terms of the quality of the information retrieved. To validate the utility of the classification system, we have defined a traffic scenario where a driving assistance system sends recommendations to drivers, such as adjusting speed or choosing alternative routes, allowing them to optimize traffic and to improve their driving experience. Most drivers who were recommended a speed adjustment or an alternative route experienced a gain in time.

The rest of the paper is organized as follows. In Section 2, we review the work in the field of driver behavior feature identification. In Section 3, we explain the factors affecting driver behavior we use in our work. Section 4 presents the description of the proposed Fuzzy System. Section 5 is devoted to the experimental evaluation. Finally, Section 6 summarizes the conclusion and upcoming work.

## 2. State-of-the-Art on Driver Behavior Feature Identification

The construction of identification models and the driver behavior classification are the two main lines of research related to our work.

The previous works on driver behavior modeling are generally based on Neural Networks [7,8], Hidden Markov Models [9,10], Fuzzy Control Theory [11,12], and Gaussian Mixture Models [13,14]. Regarding classification, it is necessary to group the different features that comprise driver behavior. Most existing works addressing feature classification have been focused on the K-means algorithm, Neural Networks, and Fuzzy Control Theory.

There are many clustering approaches based on the K-means algorithm. The work in [15] focuses on the influence of driver behavior on traffic emissions. They use a K-means clustering algorithm for classifying drivers into different profiles considering acceleration and speed data. In [16], the authors present a clustering approach based on K-means for driving style classification, with the final aim of providing a driver scoring in different road types.

Among the works inspired on Neural Networks, we can mention [7], where authors propose an intelligent driving diagnosis system and a classifier. The classifier can categorize the drivers into two groups: aggressive and moderate.

A method to judge a driving style according to energy efficiency, using recurrent neural networks and GPS data, is presented in [17]. The aim is to give drivers advice on how to optimize their driving style in order to reduce energy consumption on a road trip. Other similar works are [18,19], where they use Deep Convolutional Neural Networks to classify driver behavior. Finally, ref. [20] combines neural networks with Random Forest for dangerous driving classification.

Among the works that use Fuzzy Logic, we can mention the one presented in [21]. Their system deploys a fuzzy-set qualitative comparative study to survey data to obtain profiles of drivers who frequently use smartphones while driving. They identify three personality profiles: non-neurotic driver, extraverted-open driver, and conscientious driver. In a similar way, ref. [22] classify driving styles using neuro-fuzzy modeling, aimed to classify the driving behaviors by matching to fuzzy patterns extracted from smartphone sensors data. The work in [23] uses the fuzzy logic-based classification for promoting eco-driving, considering the driver behavior and the effects of some environmental contexts. Finally, in [24], we presented a preliminary design of a Fuzzy Rule-Based System for driver classification, according to the behavior. The system was designed to be integrated into an Intelligent Transportation System aimed at routing optimization and some other tasks for improving driving safety and comfort.

Other approaches propose using supervised classification methods instead of Fuzzy Logic algorithms, such as Bayesian classification [25]. However, it requires detailed prior knowledge for the different parameters to be classified. The work in [26] proposes the use of a Naive Bayes classification method for monitoring driving behavior. Using accelerometer sensor data, the system can detect events such as turns, accelerations, and decelerations and then use the Naïve Bayes method to classify the driver behavior into three categories: normal, defensive, and aggressive.

Some recent works use onboard image sensors to detect driver behavior. The paper in [27] uses convolutional neural networks for the detection of driver distractions, and they use deep learning to process image details. The method uses features extracted from images to make the classification. The system can recognize different situations: safe driving; use of the phone, for either texting or call, and distinguishing the hand the driver uses for the action; handling the radio; looking behind; hairstyle and makeup adjustments; and when drivers drink something or talk to passengers. Another sensor-based framework for driver behavior monitoring is [28]. They use information about head movement (acquired from acceleration sensors) and about the deviation of the vehicle across the lane (using the data from onboard image sensors). Considering these data, the system categorizes driving states and sends alert messages in case of dangerous behavior to nearby vehicles and pedestrians.

Despite their great flexibility, efficiency, and fault tolerance, Neural Networks require a complex learning process, which needs a large amount of data and generally takes a long time. Furthermore, neural networks have some limitations, such as fixed sized inputs and lack of memory when used for pattern recognition tasks [29]. Fuzzy Logic, on the contrary, is an easy-to-implement option offering good results in many complex nonlinear problems with vague and imprecise knowledge.

From the above discussion, we can see that most of the works found in the state-of-the-art fail in environments with a lack of input data, which constitutes an important limitation for their potential application in real environments. In addition, there is no previous approach, to our knowledge, based on a knowledge model providing the level of expressiveness and flexibility that an ontology provides.

Our work aims to fill this gap by providing an ontology-based knowledge model, expressive enough to cover all the particularities of driver behavior related to traffic-specific situations and constraints, and by providing an inference system which is able to deal effectively with the absence of input data and the presence of uncertainty.

## 3. Factors Affecting Driver Behavior

Driving pattern is a complex phenomenon, influenced by several variables such as the driver, street environment, traffic flow, and car type [30]. The elements that affect the driver’s behavior could be vehicle-related, such as the state of the different vehicle parts; environment-related, such as the road, traffic signs, and weather conditions; and driver-related. The driver-related ones are the human factors that affect the driver behavior, such as gender, age, and psychophysical state. Some human characteristics may yield a reduction in the alertness level while driving, such as age, eating, drugs or alcohol consumption, circadian rhythms, or any disease [31]. Social and economic factors, such as educational level or job situation, may also affect driver behavior.

Among all the factor affecting driver behavior, we have selected the one which have a greater impact on driver speed, since speed can have a huge impact on the risk of traffic accidents for several reasons. Driving at high speed can raise the risk of accidents by increasing driver reaction time to hazards in the road environment. Although there are other factors too, such as cornering, speed is still one of the main predictors of harmful traffic events. Therefore, for this work we have selected the following factors, due to their effect in driving speed:

- Age and gender: There are some studies about the relationship between the gender and the driver age group with the road crashes. Some studies, such as [32], state that the percentage of men involved in traffic accidents is greater than women, independently of age. Regarding age groups, the same study shows that most traffic accidents involve people under 25 years, while a low percentage of accidents occurs in people over the age of 70, taking into account that the percentage of drivers over 70 years is much lower than in other age groups.
- Road environment: The road environment can affect driver’s perception through some implicit information, such as the peripheral visual field, and explicit information, such as traffic signs. Road geometry is a factor that can affect the speed choice and speed perception. It is defined by the surface characteristics, the number of lanes, the width of each lane, the road curvature, the delineation, etc. Another factor is roadside development and objects near the road. Objects located very close to the road may force drivers to react, adapting their speed to the current situation. Other visible objects, even if they are far from the road, can also affect speed. For instance, the presence of trees, or buildings may cause drivers to slow down on those roads [33]. Objects can also cause driver distractions and overload.
- Temporary factors: Temporary factors may also affect driver behavior. The time of the day can affect speed, since usually less visual information is available at night than during the day. The presence of other road users may also affect driver speed, since drivers have to take into account the behavior of other cars, pedestrians, etc. There are other temporary factors such as parked cars, weather conditions [33], roadworks, or any special events that affect the traffic on specific roads [34].

## 4. Architecture of the System for Driver Behavior Classification

**Figure 1:**
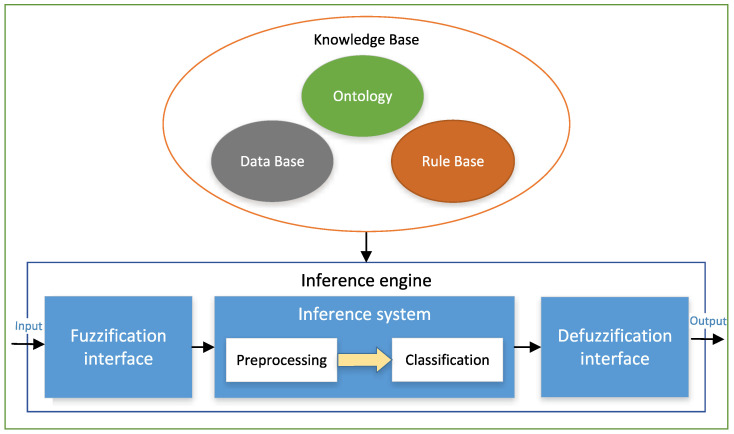
Structure of the proposed classification system.

The proposed Fuzzy System for the driver behavior classification is based on the Mamdani approach [35] and has been developed in Java. Figure 1 shows the system’s architecture, which is composed of two main parts: the inference engine and the knowledge base. The different parts of the inference engine and the knowledge base will be explained below.

### 4.1. The Inference Engine

The principal role of the inference engine in a Fuzzy System is to apply the rulebase over the symbolic data received as input and to produce the conclusion as an output [36]. The inference engine is composed by the inference system and the fuzzification/defuzzification interfaces.

1. The fuzzification interface allows the fuzzy rule-based system to translate crisp inputs to their corresponding values in the fuzzy sets with which the inference system operates. Fuzzification is the process of determining the membership degree of an input value to a specific fuzzy set. This process is performed via membership functions.
2. The inference system generates fuzzy outputs from fuzzy inputs obtained from the fuzzification interface, according to the rulebase. Each rule is evaluated using a conjunction operator and an implication operator. The classic conjunction and implication operators used in fuzzy logic are t-norm functions, due to their properties: monotonicity, associativity, commutativity, and having unity as the neutral element. Among the best known t-norms are the minimum and product functions. In this work, we have chosen the minimum as conjunction and implication operator, since it is the largest of the t-norms and therefore the most used in this type of system.
  The inference system is composed of two levels: the pre-processing level, which process the raw data to obtain the inputs for the classification, and the classification level, which obtains the final driver profile.
3. The defuzzification interface aggregates the information from the fuzzy sets and converts it into a crisp value (inference). Defuzzification can be performed in different modes, considering the order of aggregation and inference operations. In this work, we choose the mode FATI (First Aggregate, Then Infer) for defuzzification. Aggregation incorporates the individual fuzzy sets to a global one, using an aggregation operator. The aggregation operator is usually a t-conorm. This is due to the properties of this type of function: monotonicity, associativity, commutativity, and 0 as the neutral element. Examples of t-conorms are the maximum and sum functions. Maximum is the function for union in classical sets, so it is also the typical aggregation operator in fuzzy systems. In this work, we used the maximum t-conorm as aggregation operator for defuzzification.
  The second step consists of transforming the fuzzy set into a crisp value, using a defuzzification method. There is a great variety of defuzzification methods [37]. The most widely used are the centroid- and the maximum-based defuzzification techniques. In the case of the centroid technique, the crisp output value is computed as the center of the area of the membership function for the fuzzy value. In the maximum-based methods, the crisp output value is chosen from the values at which the membership degree is maximum. Depending on how this value is obtained from the maximum membership value set, there are some variations, such as the mean of maxima, the smallest maxima, and the largest maxima.
  For the selection of the defuzzification method, we tested the system with six of the most common defuzzification algorithms based on both the centroid and maximum approaches. These methods were: center of area (COA), fuzzy mean (FM), weighted fuzzy mean (WFM), first of maxima (FOM), last of maxima (LOM), and mean of maxima (MOM) [37]. In the case of centroid-based methods, the results are more disperse. This means that more membership functions are needed for more accurate classification. In contrast, in the maximum-based methods, as only the values with maximum membership are considered, the resulting sets of values are more delimited and therefore a more accurate result is obtained using fewer membership functions.
  From these results, we concluded that the more suitable approach for our system was the maximum-based one. Thus, we choose the last of maxima (LOM) as the defuzzification algorithm. In this method, the crisp output value selected is the largest of the values belonging to the set of values with the highest membership degree.

The absence of data can affect the operation of the inference engine, giving erroneous results when some input data is not provided. To deal with the absence of input data, in this work, we take advantage of the properties of the minimum t-norm, used as a conjunction operator in the inference system. One of the properties of all t-norms is that the neutral value is 1, so min (x,1)=x. As the inference system applies the conjunction operator (minimum) between the membership degrees when evaluating the rules, we use the value 1 (upper limit of membership) as the membership degree in case of missing values. Thereby, we ensure that the absence of data does not penalize the result because the system “ignores” missing values.

### 4.2. The Ontology

**Figure 2:**
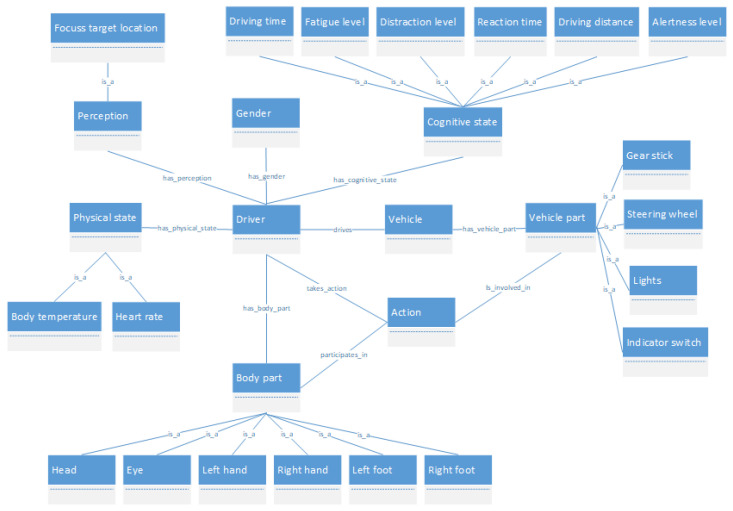
Part of the driver behavior ontology with a selection of the main classes and relations defined for modeling the driver behavior.

The most important element regarding the knowledge base of the system is the ontology. Taking into account the driver behavior model presented in [38], we have developed a driver behavior ontology that covers different entities and relationships within the driver behavior domain. This ontology is connected with a road traffic ontology [39] that contains some entities and rules related to the traffic domain. Figure 2 presents a partial view of the behavior ontology proposed.

Most of the concepts in the behavior ontology spin around a principal one, which represents the driver. This concept has a set of attributes such as age and gender. In addition, it includes characteristics related to the physical state, such as the heart rate and body temperature. Moreover, there are characteristics regarding perception, which cover a set of concepts dealing with infrastructure elements the driver can see in different points of the route. Finally, there are characteristics related to the cognitive state, encompassing concepts as reaction time or alertness level, among others.

Another relevant set of concepts modeled in the ontology is deal with the different actions performed by the driver and their relationships with the corresponding vehicle parts and driver body parts involved in the action. In addition to the act of driving, the action is considered a crucial connection between the driver and the vehicle. Taking this into account, we have defined a set of actions that the driver could execute, such as accelerating, braking, steering movement, etc. For each action, we have associated the vehicle parts and the driver body parts used for their execution. For example, the steering movement should involve the left and right hands of the driver and the steering wheel of the car.

### 4.3. The Database

The database used in this work has been developed from the results provided by [40]. They first collected data from a virtual 3D driving simulator and then proposed a driver behavior model and a rule base that describes the normal actions that the driver should perform in various traffic scenarios, such as up and down hills, around curves, etc. The simulator has a widescreen, which allows for displaying a very realistic environment. This allows the simulator to collect driving data from humans in a very close-to-real-life scenario. In this work, we use the same set of rules to a priori characterize the “normal” driver behavior in the predefined routes according to the use of the brake and accelerator.

Then, we compare the current behavior with the “normal” model to obtain the percentage of times an unsuitable action occurs. In Table 1, as in [40], we show a small set of rules related to the normal use of the accelerator in different traffic situations in predefined routes. As we can see in the table, if the vehicle is on a curve (or it is approaching a curve), it is normal for the driver to release the accelerator. The driver also releases the accelerator if it is on (or it is approaching) a downward slope and also if the desired speed has been exceeded. On the contrary, the driver accelerates if it is moving straight, on an uphill slope, or under the desired speed.

### 4.4. System Parameters

The system classifies drivers into different profiles taking into account the frequency of the use of the accelerator and brake and the speed variation.

**Figure 3:**
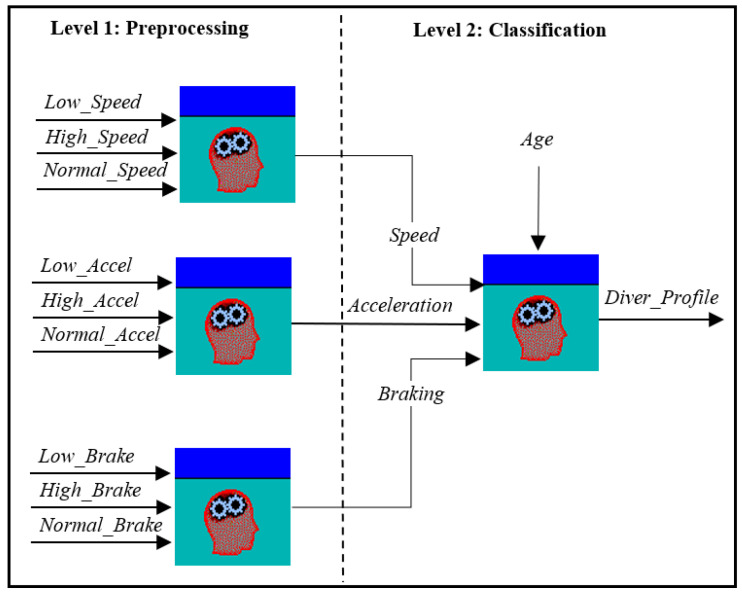
System parameters.

The fuzzy variables were divided into fuzzy sets with different membership functions. In this work, we first analyzed the data distribution for the different variables to determine the best suitable function to model them. In all cases, we found a high level of randomness in the data distribution between an upper and a lower bound. Because of this, we decided to use the typical triangular and trapezoidal-shaped membership functions, distributing the fuzzy sets according to our data. For this system, we have used an evolution of the parameter set we proposed in [24]. Figure 3 shows the relationship between the system variables, which are processed at the two levels of inference. At the preprocessing level, raw data related to speed, accelerator use, and brake use is received as input. These variables are combined using fuzzy rules to obtain the fuzzy input parameters of the main level, which is the classification level. The parameters of the classification level are described below.

**Figure 4:**
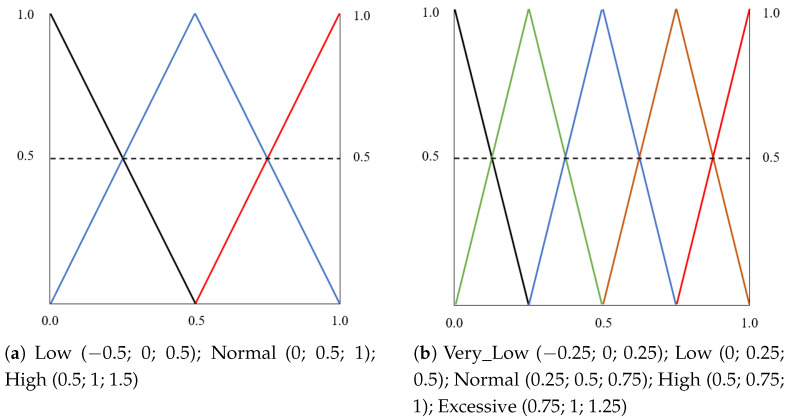
Membership functions and fuzzy sets for (**a**) the % of times driving under the minimum, over the maximum, and within the right speed limits; (**b**) the final speed indicator.

**Figure 5:**
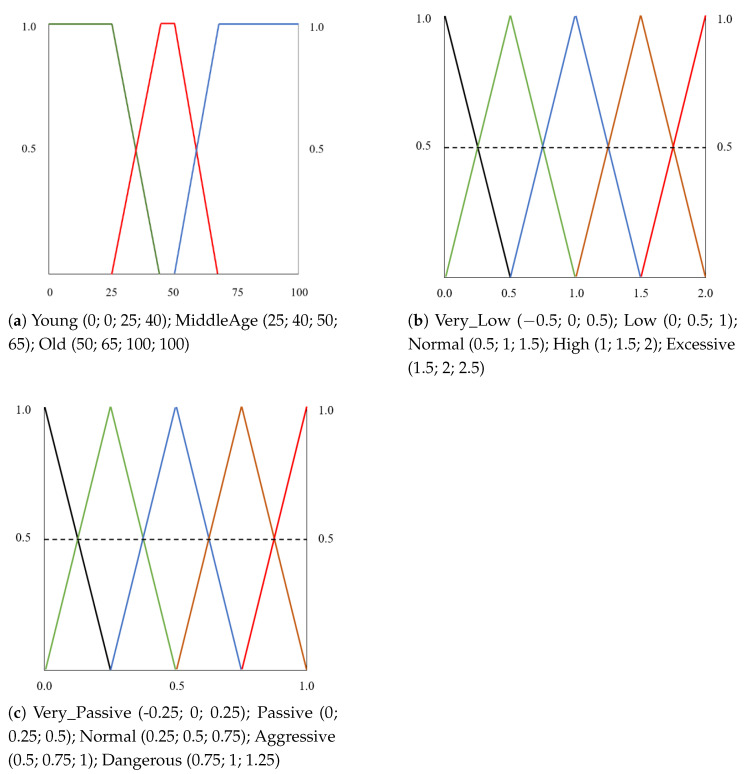
Definition of membership functions and fuzzy sets. (**a**) Trapezoidal membership functions and fuzzy sets representing the age. (**b**) Five equally spaced triangular-shaped membership functions and fuzzy sets representing acceleration and braking. (**c**) Five equally spaced triangular-shaped membership functions and fuzzy sets representing the driver profile.

- Speed: Input variable, which describes the speed indicator. This parameter is computed as a fuzzy combination of three input values: (a) Low_Speed: percentage of times driving under the minimum speed limit; (b) High_Speed: percentage of times driving over the maximum speed limit; and (c) Normal_Speed: percentage of times driving within the correct speed limits. The fuzzy sets were designed using triangular-shaped membership functions representing the variables involved in the speed indicator computation (preprocessing level). Figure 4a shows the three fuzzy sets and membership functions that represent the input values for the speed indicator calculation. For the speed indicator output, we have defined five triangular membership functions (Figure 4b).
- Age: Describes the age of the driver, which is a well-known and widely used parameter, usually modeled using trapezoidal membership functions according to the age groups of the problem under study. Figure 5a shows the membership functions and fuzzy sets defined to describe the age in our setting.
- Acceleration and braking: Acceleration describes the way the accelerator pedal is used by the driver throughout the route. This parameter is computed as a fuzzy combination of three input values: (a) Low_Accel: percentage of times in which the driver should accelerate and does not accelerate; (b) High_Accel: percentage of times in which the driver should not accelerate and accelerates; and (c) Normal_Accel: percentage of times in which the driver makes correct use of the accelerator. Braking represents the way the brake is used by the driver throughout the route. This parameter is computed as a fuzzy combination of three input values: (a) Low_Brake: percentage of times in which the driver should brake and does not brake; (b) High_Brake: percentage of times in which the driver should not brake and brakes; (c) Normal_Brake: percentage of times in which the driver makes correct use of the brake. In both cases, the input variables of the preprocessing level were designed using triangular-shaped membership functions as presented in Figure 4a. For the acceleration and speed output, five fuzzy sets with triangular-shaped membership functions were defined. Figure 5b presents the membership functions and fuzzy sets defined to describe the acceleration and braking parameters.
- Driver profile: the output variable of the fuzzy system. It represents the driver profile. For this variable, 5 equally spaced, triangular-shaped membership functions were defined. Figure 5c presents the membership functions and fuzzy sets defined for the driver profile. The driver profile is classified into five categories: *Very Passive*, *Passive*, *Normal*, *Aggressive*, and *Dangerous*.

### 4.5. The Rulebase

The rulebase was deduced using a dataset from heuristics of experts. The selection of experts was made up of a group of new and experienced drivers, belonging to different age groups. First, the experts manually classified the behavior of the drivers from the data collected in the simulator. Then, the resultant dataset was partitioned with a fold Cross Validation method and was used in the genetic algorithm THRIFT [41] for the automatic learning of fuzzy rules of the Mamdani type. The fitness function used was the Mean Square Error (MSE), which is expressed as follows:

(1) $$MSE=\frac{1}{n}\sum_{i=1}^{n}{\left(Y-\tilde{Y}\right)}^{2}$$

where *n* is the number of samples, *Y* is the set of observed values, and $\tilde{Y}$ is the set of estimated values. The best individuals were those that minimized MSE.

For crossover, we used the standard *two-point operator* and the *elitist* selection mechanism [42]. The input parameters of the algorithm were: Population_Size = 50, Number_of_Evaluations = 1000, Crossover_Probability = 0.6, and Mutation_Probability = 0.1. Because it is difficult to show the entire rulebase, we have selected five representative rules for the main driver behavior profiles, which we show in Table 2.

## 5. Experiments

The experiments were performed in a simulated environment to validate the performance of the driver classification system in different traffic road scenarios and to evaluate how the system can improve traffic flow on ITS. The validation phase was divided into two stages, according to these two dimensions. The results of the two stages are presented below.

### 5.1. Validating the Classification Module and the Expressiveness of the Knowledge Base

This phase is addressed to validate the driver classification system. The results obtained from the traffic simulator were analyzed by experts, who made a manual classification that was used as a reference when evaluating the system.

To validate the effectiveness of the proposed classification method, a comparison was made with two different approaches: a probabilistic classification method based on Naive Bayes, such as the one proposed in [26], and a Random Forest classifier. The classification was performed using the 10-fold cross-validation method with a dataset composed of 300 samples collected from the traffic simulator. Each record has the following 9 columns:

- Number of segments in which the user drives over the maximum speed limit;
- Number of segments in which the user drives under the minimum speed limit;
- Number of segments in which the user drives within the allowed speed limits;
- Number of segments in which the driver should accelerate and does not accelerate;
- Number of segments in which the driver should not accelerate and accelerates;
- Number of segments in which the driver makes correct use of the accelerator;
- Number of segments in which the driver should brake and does not brake;
- Number of segments in which the driver should not brake and brakes;
- Number of segments in which the driver makes correct use of the brake.

In the case of a Random Forest classifier, the system creates multiple random decision trees and subsequently aggregates the results to obtain a single output. As the accuracy of this algorithm depends largely on the number of trees used, we have applied the algorithm varying the number of trees from 10 to 500, to select the best configuration. The lowest error rate was obtained with a configuration made up of 50 trees for the five features.

**Figure 6:**
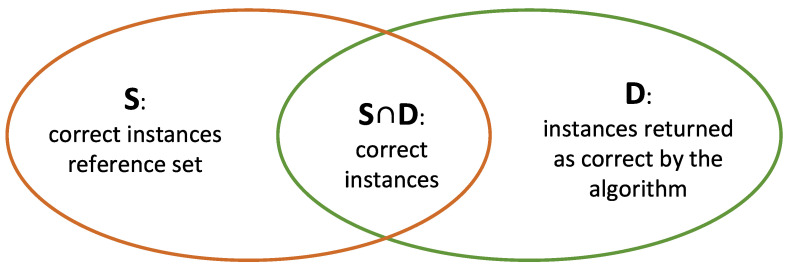
Parameters involved in *Precision* and *Recall* computation.

The evaluation of the classification systems was carried out using MSE and also *Precision*, *Recall*, and *F-Measure* (*F1*), which represents accuracy and completeness [43]. MSE was computed using Equation (1), considering the membership degree values observed for the different fuzzy sets of the output variable and the expected values in the reference set. For the *Precision*, *Recall*, and *F-measure* calculation, we use the set of correct reference instances (*S*) and the set of instances returned by the algorithm as accurate (*D*), as seen in Figure 6.

These measures are defined below:

- *Precision* represents accuracy. It is given as the ratio between correct instances, and those retrieved as correct by the algorithm [42]. Equation (2):
  (2) $$P\left(D,S\right)=\frac{\left|S\cap D\right|}{\left|D\right|};$$
- *Recall* represents the fullness. It is given as the ratio between correct instances and those defined as correct in the reference set [43]. Equation (3):
  (3) $$R\left(D,S\right)=\frac{\left|S\cap D\right|}{\left|S\right|};$$
- *F1* is considered an aggregation of both *precision* (*P*) and *recall* (*R*) [43]. The weight of each measure depends of a factor α between 0 and 1. *F1* is defined by Equation (4):

(4) $$F_{\alpha }\left(D,S\right)=\frac{P(D,S)R(D,S)}{\left(1-\alpha )P(D,S)+\alpha R(D,S\right)}.$$

The results obtained are shown in Table 3. It presents the MSE, and the average of *precision*, *recall*, and *F1* values obtained on all the iterations. In the case of *F1*, we have defined α=0.5. As we can see in the table, the MSE value in the fuzzy classifier was lower than in the Random Forest and Naive Bayes classifiers. Considering the average of *Precision*, *Recall*, and *F1*, the results of the fuzzy classifier also outperform the other approaches.

**Figure 7:**
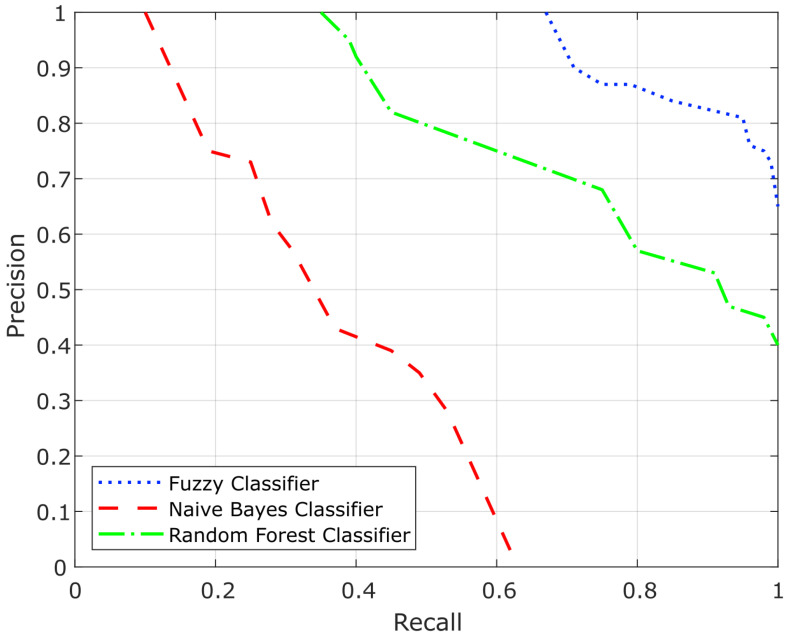
*Precision* vs. *Recall* curves for the Fuzzy classifier, the Random Forest classifier, and the Naive Bayes classifier.

Figure 7 shows the *precision–recall* curves for the three methods. In the curves, we observe how the *precision* values decrease as the *recall* values increase. We can see that the Bayesian classifier yields the lowest values for *precision* and *recall*. This is due to the fact that this approach assigns 0 probability to the categories of the test variables which are not observed in the training data. This is a clear disadvantage of this approach compared to the random forest and fuzzy logic approaches. Another characteristic of the Bayesian classifier is that it assumes completely independent predictors. Although this implies that the Bayesian classifier requires little training data, it makes the model unrealistic, and it is a limitation that affects *precision* and *recall*.

The Random Forest classifier yielded a lower error rate and better *precision* and *recall* values than the Bayesian classifier. It proposed a more realistic model, which is less affected by the absence of data thanks to the use of bagging as the ensemble method. However, the results are worse than those of the fuzzy classifier. This is mainly because the random forest requires much more training data to make good classifications. This implies a higher computational cost.

On the other hand, the method based on Fuzzy Logic achieves the highest *precision* and *recall*. It shows that a Fuzzy Logic approach could be more suitable for this kind of problem with high uncertainty and little training data than a probabilistic approach or a random forest approach.

In evaluating the expressiveness of the knowledge base, we use the mechanism of reasoning implemented in the ontology through the reasoner Pellet [44]. The reasoner allows us to infer new knowledge through rules and, at the same time, to check the consistency of the ontology. Two sets of queries were defined in SPARQL [45]: the first (Q1) to evaluate the information retrieval and the consistency of the knowledge model and the second one (Q2) to verify the expressiveness and consistency of the traffic ontology in different scenarios. To validate both sets of queries, the results were compared with a reference result set obtained through questionnaires to experts.

The set Q1 is composed of 20 queries, divided into four categories: queries related to perception, queries related to physical state, queries related to cognitive state, and queries related to actions. The set Q2 is composed of 36 queries, divided into three categories: veicle-related queries, infrastructure-related queries, and traffic-related queries. Due to space reasons, we cannot show all the queries, so we have selected a short example. The following query returns the list of vehicles on a given road:

{SELECT}  ?Vehicle ?Color ?Model ?Brand ?Registration_Number ?Type ?Lane

WHERE

{ ?Vehicle rdf:type Traffic_ontology:vehicle .

?Vehicle Traffic_ontology:isOnRoad Traffic_ontology:road1 .

?Vehicle Traffic_ontology:has_color ?Color .

?Vehicle Traffic_ontology:has_model ?Model .

?Vehicle Traffic_ontology:has_brand ?Brand .

?Vehicle Traffic_ontology:has_registration ?Registration_Number .

?Vehicle Traffic_ontology:has_vehicle_type ?Type .

?Vehicle Traffic_ontology:isOnLane ?Lane

}

The results the validation of the two sets of queries to checking the expressiveness of the knowledge base are shown in Table 4. The experiments were setup considering *precision*, *recall*, and *F1*. In the case of *F1*, we defined α = 0.5.

For both query sets, the values obtained for the three measures are satisfactory. As we can see in the table, for both reasoning experiments, the values of *recall* were better than the *precision* ones. That means that in both experiments, we have retrieved all the expected information, but a bit more information than we expected. Thus, we can improve the exactness of the retrieval with the addition of more reasoning rules that provide more accuracy in the query responses.

### 5.2. Traffic Flow Optimization Validation

In this phase, we have simulated different traffic scenarios through a multi-agent system, using JADE [46]. We estimated the arrival time to the destination, taking into account the traffic conditions of the current route, the possible alternative routes and the behavior profile of the driver. Based on that estimation, three different options are suggested to the driver: to take an alternative route, to increase the speed, or to decrease the speed.

The experiments were carried out on a simulated map, where we defined 30 routes in a radius of approximately 15 km. First, the driver profile is obtained using the proposed classification system with a predefined set of randomly assigned behavior parameters (speed, acceleration, age, etc.). A route is then randomly assigned to each vehicle. Each route is represented in the traffic ontology as a vector with a series of points, where each point is an intersection. Each point of the route has its location on the map and the action that should be taken to go to the next point (e.g., go straight, turn left, or turn right). According to the traffic rules defined in the ontology, it is possible to know whether a movement is allowed or not.

The next step is to calculate the travel time for each vehicle for two situations: the driver follows the actions recommended by the system ($T_{2}$) or the driver ignores the system recommendations ($T_{1}$). Then, for each vehicle, we compute the time variation for the two situations (ΔT) through Equation (5):

(5) $$\Delta T=T_{1}-T_{2}\left\{\begin{array}{c} >0,\mathrm{time}\;\mathrm{gain}\\ =0,\mathrm{no}\;\mathrm{change}\\ <0,\mathrm{time}\;\mathrm{loss} \end{array}\right.$$

If ΔT>0, the recommendations yielded a time gain to the vehicle; if ΔT<0, the recommendations yielded a time loss to the vehicle; and if ΔT=0, the recommendations had no effect on the vehicle travel time.

**Figure 8:**
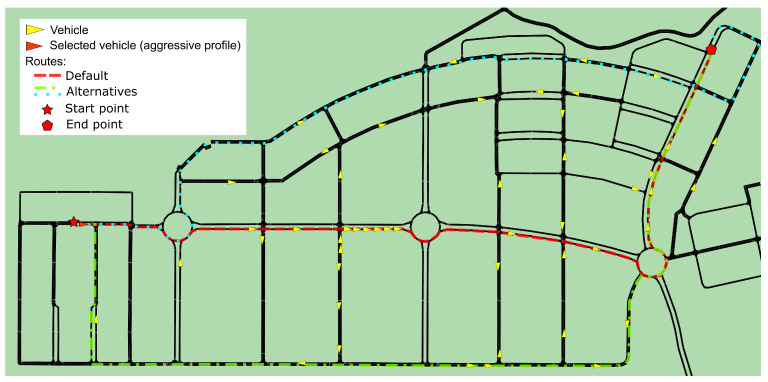
Example of a route optimization case using the fuzzy classification system.

Finally, we compared the results obtained in both cases. Three sets of experiments were performed, varying the number of vehicles: the first in a range between 50 and 150 vehicles; the second between 150 and 300 vehicles; and the third between 300 and 500 vehicles. Figure 8 shows an example, with 50 vehicles located on the map. Vehicles are shown as yellow triangles, which indicate the direction of their movement. For a better understanding, we have chosen a specific vehicle, and we represent only the routes associated with the selected vehicle. The default route for this vehicle is represented by a red dashed lines (a red star represents the start point and a red pentagon represents the endpoint). Considering the level of congestion and the driver profile (aggressive in this case), the system suggests two alternative routes (blue and green dashed lines) to save time.

Table 5 presents the result of the tests performed in this stage. The columns of the table are detailed below:

- Vehicles: represents the number of vehicles used in the experiment;
- Alternative routes suggested: shows the percentage of cases in which alternative routes were suggested, considering the driver profile;
- Time gain: the percentage of cases where vehicles gained time after taking an alternative route;
- Time loss: the percentage of cases where the vehicles suffered a time loss after taking an alternative route;
- No change of time: the percentage of cases in which no gain or loss of time was experienced after taking an alternative route;
- Speed reduction suggested: the percentage of cases in which speed reduction was suggested;
- Speed increasing suggested: the percentage of cases in which speed increasing was suggested.

There were fewer suggested alternative routes when more vehicles were included in the scenario. This is because when there is greater congestion, the time to destination estimate for an alternative route may not be better than for the current route. In those cases, the recommendation is not to change the route. In cases where a speed decrease was recommended, vehicles experienced a time loss, while, on the other hand, vehicles that increased speed gained time. However, we consider that these increases and reductions in speed, beyond influencing the time to destination, are essential to guarantee road safety. For that reason, we do not include the losses and gains of time caused by speed changes in the table. As we can seen in the table, most of the vehicles that took the suggested alternative route experienced a time gain. On the other hand, time loss was observed in cases where the suggested alternative routes had a higher volume of traffic. In some of the cases in which the vehicles did not experience any time gain or loss, the change of route contributed to reducing the congestion in the current route, allowing more fluidity of traffic.

## 6. Conclusions and Upcoming Work

This work proposes a Fuzzy System that addresses driver classification into different profiles depending on their behavior. For the classification, we take into account variables related to the driver behavior such as the acceleration, braking, and speed variation along different routes in a traffic simulator.

The system helps to prevent harmful transportation situations, such as road crashes, and traffic jams. This is achieved by making suggestions to the driver to take different actions, such as taking alternative routes or changing the speed. Suggestions are made considering the driver’s behavior profile and the congestion level of the routes.

Several experiments were carried out divided into two stages: first, to evaluate the effectiveness of the classification system separately and then to validate the full system. The results obtained in the evaluation of the classification system were satisfactory in terms of *precision* and *recall*. Regarding the tests of the full system, the results show that a high percentage of the vehicles that followed the suggestion of changing the route experienced a gain in time. In general, these results demonstrate the effectiveness of the system to improve traffic flow and optimize routes.

As immediate future work, we want to address some of the limitations of the present work. We have found that *precision* can be improved by including other variables that provide more information and thus contribute to the system accuracy. More specifically, we are planning to include more variables into the system, such as road signs, road conditions, weather conditions, fuel costs, etc. To improve the *precision* of the classification system, we also plan to consider more parameters, such as physical condition, attention level, and time driving, among others related to the driver status. Another limitation is the fact that we only took into account the level of congestion and speed for the estimation of the arrival time when validating new route suggestions. We are now considering the possibility of validating the classification system in real driving scenarios and expanding the verification of the results through qualitative surveys of different users.

## Figures and Tables

**Table 1:** Rule set for “normal” use of the accelerator in different road situations.

Rule	Antecedent	Consequent
1	Car(x) ∧ Curve(y) ∧ On(x, y)	Release_Accelerator
2	Car(x) ∧ Straight(y) ∧ On(y, x)	Accelerate
3	Car(x) ∧ HillUp(y) ∧ On(y, x)	Accelerate
4	Car(x) ∧ HillDown(y) ∧ On(y, x)	Release_Accelerator
5	Car(x) ∧ Curve(y) ∧ InSight(y, x)	Release_Accelerator
6	Car(x) ∧ Straight(y) ∧ InSight(y, x)	Accelerate
7	Car(x) ∧ HillUp(y) ∧ InSight(y, x)	Accelerate
8	Car(x) ∧ HillDown(y) ∧ InSight(y, x)	Release_Accelerator
9	Car(x) ∧ OverSpeed(x)	Release_Accelerator
10	Car(x) ∧ UnderSpeed(x)	Accelerate

**Table 2:** Sample rules for the driver classification system.

Rule	Antecedent	Consequent (Profile)
1	Age (“old”) ∧ Accelerator (“very_low”) ∧ Brake (“very_high”) ∧ Speed (“very_low”)	Very passive
2	Age (“old”) ∧ Accelerator (“low”) ∧ Brake (“high”) ∧ Speed (“low”)	Passive
3	Age (“Middle”) ∧ Accelerator (“normal”) ∧ Brake (“normal”) ∧ Speed (“normal”)	Normal
4	Age (“young”) ∧ Accelerator (“high”) ∧ Brake (“low”) ∧ Speed (“high”)	Aggressive
5	Age (“young”) ∧ Accelerator (“excessive”) ∧ Brake (“very_low”) ∧ Speed (“excessive”)	Dangerous

**Table 3:** Experimental results. Classification system evaluation.

Classification Method	MSE	Precision	Recall	F1
Fuzzy classifier	0.17	0.82	0.87	0.84
Random Forest classifier	0.23	0.68	0.70	0.69
Naive Bayes classifier	0.36	0.51	0.36	0.42

**Table 4:** Experimental results. Knowledge base. Expressiveness validation.

Test	*Precision*	*Recall*	*F1*
**Q1 (Driver behavior model)**	**0.78**	**0.99**	**0.87**
Q1.1 (Perception)	0.80	0.96	0.87
Q1.2 (Physical state)	0.75	1	0.86
Q1.3 (Cognitive state)	0.77	0.98	0.86
Q1.4 (Actions)	0.78	1	0.88
**Q2 (Traffic ontology)**	**0.84**	**0.95**	**0.89**
Q2.1 (Vehicles)	0.85	0.93	0.89
Q2.2 (Infrastructure)	0.92	0.95	0.93
Q2.3 (Traffic)	0.76	0.97	0.85

**Table 5:** Experimental results (% cases). Simulation on traffic scenarios.

Vehicles	Alternative Routes Suggested	Time Gain	Time Loss	No Change of Time	Speed Reduction Suggested	Speed Increasing Suggested
50–150	14.5%	82.7%	7%	10.3%	5.2%	2.1%
150–300	10.8%	60.4%	12.5%	27.1%	3.7%	1.3%
300–500	7.3%	56.1%	13.7%	30.2%	3.3%	2.9%

## Data Availability

Not applicable.

## Abbreviations

The following abbreviations are used in this manuscript:

COA	Center of Area
FATI	First Aggregate, Then Infer
FM	Fuzzy Mean
FOM	First of Maxima
ITS	Intelligent Transportation Systems
JADE	JAVA Agent DEvelopment Framework
LOM	Last of Maxima
MOM	Mean of Maxima
MSE	Mean Square Error
WFM	Weighted Fuzzy Mean
